# NLRP3-Induced NETosis: A Potential Therapeutic Target for Ischemic Thrombotic Diseases?

**DOI:** 10.3390/cells12232709

**Published:** 2023-11-26

**Authors:** Rahul Kumar, Gokul Patil, Sanjana Dayal

**Affiliations:** 1 Department of Internal Medicine, University of Iowa Carver College of Medicine, Iowa City, IA 52242, USA; rkumar2@gitam.edu (R.K.); gokul-patil@uiowa.edu (G.P.); 2 Department of Biotechnology, GITAM School of Sciences, GITAM (Deemed to be) University, Visakhapatnam 530045, India; 3 Holden Comprehensive Cancer Center, University of Iowa Carver College of Medicine, Iowa City, IA 52242, USA; 4 Iowa City VA Healthcare System, Iowa City, IA 52246, USA

**Keywords:** thrombosis, NLRP3, inflammasome, NETs, NETosis

## Abstract

Ischemic thrombotic disease, characterized by the formation of obstructive blood clots within arteries or veins, is a condition associated with life-threatening events, such as stroke, myocardial infarction, deep vein thrombosis, and pulmonary embolism. The conventional therapeutic strategy relies on treatments with anticoagulants that unfortunately pose an inherent risk of bleeding complications. These anticoagulants primarily target clotting factors, often overlooking upstream events, including the release of neutrophil extracellular traps (NETs). Neutrophils are integral components of the innate immune system, traditionally known for their role in combating pathogens through NET formation. Emerging evidence has now revealed that NETs contribute to a prothrombotic milieu by promoting platelet activation, increasing thrombin generation, and providing a scaffold for clot formation. Additionally, NET components enhance clot stability and resistance to fibrinolysis. Clinical and preclinical studies have underscored the mechanistic involvement of NETs in the pathogenesis of thrombotic complications, since the clots obtained from patients and experimental models consistently exhibit the presence of NETs. Given these insights, the inhibition of NETs or NET formation is emerging as a promising therapeutic approach for ischemic thrombotic diseases. Recent investigations also implicate a role for the nucleotide-binding oligomerization domain (NOD)-like receptor family pyrin domain-containing 3 (NLRP3) inflammasome as a mediator of NETosis and thrombosis, suggesting that NLRP3 inhibition may also hold potential for mitigating thrombotic events. Therefore, future preclinical and clinical studies aimed at identifying and validating NLRP3 inhibition as a novel therapeutic intervention for thrombotic disorders are imperative.

## 1. Introduction

Ischemic thrombotic disorder, marked by the development of obstructive blood clots, poses a significant clinical challenge. While diverse risk factors contribute to the development of thrombotic disease, age emerges as a pivotal determinant in this intricate landscape [1,2,3]. Aging leads to physiological changes in the vascular and hematologic systems, rendering individuals more susceptible to thrombosis. Older individuals exhibit a higher risk for developing thrombotic complications, such as deep vein thrombosis (DVT), stroke, and myocardial infarction (MI) [4,5,6,7,8,9]. Interestingly, a graded increase in venous thrombosis has been observed with each decade of human aging [10]. Experimental models of thrombosis, including studies involving aged mice, consistently validated the heightened susceptibility to both arterial and venous thrombosis associated with advanced age [11,12,13,14,15,16]. This review discusses age and the age-associated risk factors and their contribution to ischemic thrombotic disease.

Age-related risk factors often cluster with advancing age and contribute to thrombotic disease risk. These factors include (a) comorbidities: conditions such as hypertension, diabetes, and atherosclerosis become more prevalent with age and increase the likelihood of thrombosis [17,18,19]; (b) obesity: age-related weight gain and changes in body composition can lead to obesity, which is strongly associated with thrombotic risk [20]; (c) medications: age-related medication use, including hormone replacement therapy [21], can induce thrombosis; and (d) physical inactivity: reduced physical activity in older individuals may lead to venous stasis, promoting venous thromboembolism (VTE) [22]. Ischemia-reperfusion injury (IRI) can also interact with the aging process and may exacerbate the outcomes. For instance, the impaired endothelial function seen in aging can lead to microvascular dysfunction, enhancing the risk of thrombosis during reperfusion [23]. This synergy between IRI and aging is a relatively novel area of research and needs further attention to bridge the gap in understanding the exacerbated processes.

Advancements in our understanding of the underlying pathological mechanisms have been facilitated by reliable mouse models. While several mediators come into play in the multifaceted landscape of thrombosis, this review primarily centers on neutrophil extracellular traps (NETs) and their role as a critical contributor. It may be that NETs are more important in the conditions associated with aging, such as diabetes [24,25], than with age itself [14]. Understanding the interplay between NETosis and age or age-related factors holds promise for refining diagnostic and therapeutic strategies in the future. This comprehensive review will not only describe the various age-associated ischemic conditions linked with NET release but will also elucidate the important role played by the NOD-like receptor family pyrin domain-containing 3 (NLRP3) inflammasome as an upstream mediator of NETosis. Additionally, we will explore various therapeutic strategies targeting the inhibition of NLRP3, summarizing both the natural and synthetic compounds that exhibit the potential to serve as inhibitors. Further research is warranted to gain a deeper understanding of the underlying mechanisms, paving the way for targeted interventions aimed at reducing the burden of ischemic thrombotic diseases in aging populations.

## 2. NETosis: Bridging Immune Defense to Thrombotic Triggers

**Figure 1:**
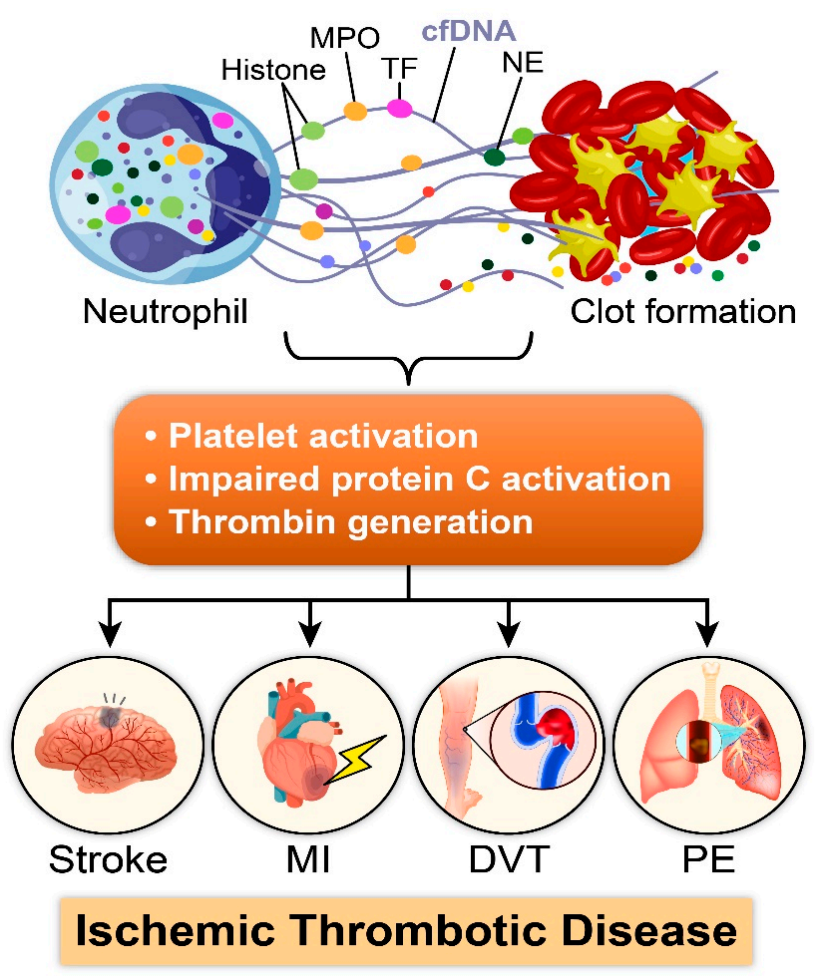
NETosis in ischemic thrombotic disease. The illustration depicts the critical role of NETosis in ischemic thrombotic disease. Neutrophil extracellular traps (NETs) released via NETosis contain DNA strands (cfDNA), histones, myeloperoxidase (MPO), tissue factors (TFs), and neutrophil-elastase (NE). NETs contribute to the formation and stabilization of thrombi via platelet activation, impaired protein C activation, and aberrant thrombin production, ultimately inducing stroke, myocardial infarction (MI), deep vein thrombosis (DVT), and pulmonary embolism (PE).

NETosis, which was first discovered by Takei et al. [26], refers to the release of NETs from neutrophils due to chromatin decondensation through the peptidyl arginine deiminase 4 (PAD-4)-mediated deimination of nuclear histones [27]. NETs contain cell-free DNA (cfDNA), histones, citrullinated histone H3 (H3Cit), elastase, and myeloperoxidase (MPO) [28]. Historically, NETosis was considered a form of innate immune response as it amplifies the local concentration of antimicrobial agents and prevents the spread of bacterial species, such as *Staphylococcus aureus*, *Salmonella typhimurium*, *Leptospira interrogans*, and *Shigella flexneri* [4,5]. However, over a decade, several studies have exemplified the prothrombotic roles of several component of NETs, including a role for cfDNA, histones or H3Cit, nucleosomes, and the tissue factor (TF) [5,6,11,12,13]. Subsequently, NETs are recognized as a pivotal contributor to the pathogenesis of numerous cardiovascular ischemic disorders (Figure 1). To provide a comprehensive understanding of this phenomenon, the following sections will delve into the mechanisms through which NETosis promotes a prothrombotic environment, shedding light on the multifaceted mechanism of NETs in thrombosis.

Further confirmation of the link between NETosis and DVT came through studies revealing that mice deficient in PAD-4, a key enzyme involved in NETosis, were protected against experimental DVT [29,30]. This suggests that the process of PAD-4-mediated NETosis plays a critical role in DVT pathogenesis. However, it is important to acknowledge that while neutrophils are the primary source of NETs, other leukocyte types, including eosinophils, basophils, and mast cells, might also contribute to extracellular trap formation under specific conditions [12,13,31,32]. These observations underscore the intricate nature of extracellular traps and their potential collaboration with other immune elements in thrombotic events.

### 2.1. Role of NETs in Thrombosis: A Multifaceted Mechanism

The critical role of NETs in thrombosis has emerged as a dynamic and multifaceted mechanism with far-reaching implications. Fuchs et al. were among the first to unveil the association between NETs and thrombosis when they demonstrated that NETs serve as a scaffold for platelet binding and aggregation [6]. In addition, NETs are involved in the inhibition of fibrinolysis through tissue plasminogen activator (tPA) inhibition [33]. Notably, extracellular DNA traps were observed within thrombi following balloon catheter occlusion in the iliac vein of baboons, providing early evidence of NET involvement in thrombus formation [6]. Subsequently, the same group confirmed these findings in an experimental DVT model in mice, using inferior vena cava (IVC) ligation [11]. 

### 2.2. Prothrombotic Effects of Individual Components of NETs

Several components of NETs possess prothrombotic activity. For example, the infusion of an unfractionated mixture of calf thymus histones was shown to exacerbate the severity of DVT following IVC ligation, where histones interfered with the generation of activated protein C (APC), leading to inadequate anticoagulation [34]. Histones were also found to mediate platelet activation [35] and platelet-dependent thrombin generation through the activation of Toll-like receptors (TLRs), such as TLR2 and TLR4 [36]. 

cfDNA, another key constituent of NETs, has been established as a promoter of thrombin generation in plasma, even in the absence of platelets. cfDNA enhances the thrombin generation potential in plasma by activating histidine-rich glycoprotein, factor XI (FXI), and factor XII (FXII) [37,38]. The studies utilizing a phorbol myristate (PMA)-induced release of NETs demonstrated an increase in thrombin generation in the plasma, a phenomenon reversed through treatment with DNase 1, implicating cfDNA in this process [14,39]. Furthermore, cfDNA and histones have been shown to confer a higher mechanical stability and fibrinolytic resistance to fibrin clots [40,41]. These observations were corroborated by studies indicating that a disruption of NETs with DNase 1 accelerates ex vivo tissue plasminogen activator (tPA)-induced thrombolysis in the thrombi collected from stroke patients [42]. Komissarov et al. further described that, at higher concentrations (1.0–20.0 μg/mL), DNA but not RNA competes with fibrin for binding to plasmin and decreases the rate of fibrinolysis [43]. While most studies suggested a role for NETs in thrombin generation, Noubouossie et al. proposed that histones and DNA, rather than intact NETs, potentiate thrombin generation [44]. The discrepant interpretation from these studies can partly be attributed to the methods of plasma preparation and raises interesting methodological questions. In the earlier studies, the plasma was prepared by performing the single centrifugation at 1500× *g* for 10 min, which might have resulted in contamination with the residual platelets or other membranous fragments [39]. However, Noubouossie et al. prepared the plasma using double centrifugation at 2500× *g* for 15 min followed by filtration using a 0.22-µm filter [44]. While acknowledging the variations in the plasma preparation methods, we recognized that this disparity offers a valuable avenue for future investigation. Beyond the methodological considerations, this opens doors to critical discussions regarding clinical implications, mechanistic insights, methodological refinements, and the potential for translational applications in treating thrombotic disorders. An exciting opportunity lies in unraveling the precise mechanisms by which histones and DNA facilitate thrombin generation in the absence of intact NETs. Such understanding may reveal novel therapeutic targets for thrombosis and related conditions. Overall, these ex vivo and in vivo studies in mice and humans establish several mechanisms via which NETs can contribute to thrombosis.

### 2.3. Platelet-Induced NETosis

A recent study suggested that platelets isolated from infarct-related coronary arteries (IRAs) of acute ST-segment elevation myocardial infarction (STEMI) patients were able to induce NETosis [45]. It indicated that platelets collected from a prothrombotic environment possess the ability to induce NETosis, that in turn acts on the platelets to induce their aggregation and activation to initiate a positive feedback regulation. Activated platelet-induced NETosis has been also demonstrated in COVID-19 and is considered a key mediator of thrombosis due to severe acute respiratory syndrome coronavirus 2 (SARS-CoV-2) infection [46]. For instance, P-selectin and α_IIb_β3, which are expressed on activated platelets, can induce NETosis via its interaction with P-selectin glycoprotein ligand-1 (PSGL-1) [47] and SLC44A2 expressed on neutrophils [48], respectively. In fact, a polymorphic site within the SLC44A2 (rs2288904-A) gene is known to influence the susceptibility to thrombosis [49,50,51]. Audriller et al. demonstrated that the activation of platelets using a thrombin receptor-activating peptide (TRAP) [52] and lipopolysaccharide (LPS) [53] can lead to robust NET formation. Taken together, these findings suggest that platelets are important hematopoietic cells for inducing the release of NETs. 

## 3. NETosis in Disease Condition

In recent years, NETosis has emerged as a critical player in the context of aging and age-associated diseases [54]. Neutrophils from aged individuals or aged mice exhibit altered NETosis kinetics, with some reports suggesting an enhanced NET formation with age [55,56], or in aged individuals with severe vasculitis [57,58], while others indicate minimal or no change in NETosis in otherwise healthy aged mice [14]. The age-related changes in NETosis may contribute to chronic inflammation, a hallmark of aging, and may also impact the immune response to infections in the elderly. Therefore, understanding the mechanisms underlying the age-related alterations in NETosis is crucial for unraveling the complexities of aging. This section aims to explore the intricate interplay between NETosis and its contributions to age-associated ischemic diseases and will also provide a brief insight into the association of NETs in non-ischemic diseases.

### 3.1. NETosis in Acute Ischemic Conditions

This section provides a brief account of the various ischemic conditions where NETs have been implicated. Elevated plasma levels in extracellular histones have been reported in ischemic or thrombotic conditions, such as myocardial infarction [59], stroke [60,61], ischemic outcomes after angioplasty [62], and DVT [11,63]. Stakos et al. observed that neutrophils isolated from IRA aspiration in STEMI patients were more prone to the release of NETs in comparison to non-infarct-related coronary arteries and control individuals [45]. NETs were found to be constitutively present in the thrombi retrieved during endovascular therapy in patients with acute ischemic stroke [42]. However, the authors could not find any significant association between the circulating markers of NETs and the final thrombolysis in cerebral infarction (TICI) score [42]. Borissoff et al. reported that the circulating markers of NETosis were independently associated with the severity of coronary atherosclerosis, the occurrence of major adverse cardiac events, and the presence of a prothrombotic state [64]. Surprisingly, a deficiency of PAD-4 in hematopoietic cells did not display a significant impact on the progression of atheromatous plaques in hypercholesterolemic mice [65], despite detecting the presence of NETs in atherosclerotic lesions from human carotid endarterectomy tissue [65]. This may indicate that the mechanisms may differ between mice and humans. An alternative explanation could be that the process of inducing disease conditions in mice may not necessarily phenocopy human disease pathology. Retinal vein occlusion (RVO), which refers to the obstruction of the retinal venous system due to thrombus formation, has emerged as the second most common retinal vascular disorders [66,67]. Recently, Wan et al. reported that the elevated plasma levels in NETs are associated with RVO and can be exploited as a potential biomarker [68]. In summary, NETs have been identified in several ischemic conditions but their mechanistic contributions to pathological ischemia is not fully understood in most of the disease states.

### 3.2. NETosis in Cancer-Associated Thrombosis

Several tumors and cancer cells secrete a cytokine called the granulocyte colony-stimulating factor (G-CSF) [69,70,71,72,73], which exhibits the potential to induce NETosis and promote thrombosis [74]. Higher levels of circulating NET markers predicted the occurrence of DVT in cancer patients and was associated with a poor prognosis in a large cohort in the Vienna Cancer and Thrombosis Study (CATS) [75]. This was an important study since it included patients with malignancy at varied sites, such as brain, breast, bronchus, stomach, prostate etc. [75]. The elevated circulating levels of MPO-DNA was also associated with thrombosis in patients with myeloproliferative neoplasms (MPN) [76]. Similarly, histone–DNA complexes have been demonstrated in the thrombi in patients with different cancer types [77,78] and circulating H3Cit has been associated with the markers of thrombosis [77]. Consistent with these findings, we showed that the expression of circulating cfDNA and thrombin generation potential was increased in pediatric patients with acute lymphoblastic leukemia and that the treatment of plasma with DNase 1 lowered the potential for thrombin generation [79]. These findings suggest a link between NETs and thrombosis in cancer patients.

There was also in vivo evidence for NETs promoting venous thrombosis in mouse cancer models. Demers et al. [74], using murine models of lung and breast carcinoma and chronic myelogenous leukemia, demonstrated an increased sensitivity of neutrophils undergoing NETosis. Hisada et al. observed elevations of H3Cit and cfDNA in the plasma and thrombi from mice bearing human pancreatic tumors, and the administration of DNase 1 or the depletion of neutrophils reduced the thrombus size in mice bearing human tumors [80]. Similar observations have been made by others in mammary cancer [81,82,83]. Wolach et al. further extended the findings on the role of NETs in animal models of cancer-associated thrombosis to MPN represented by the Jak2^V617F^ mutation [84]. A Jak2^WT^ mouse transplanted with Jak2^V617F^ bone marrow had elevated NETs compared to the Jak2^WT^ mice and developed spontaneous pulmonary thrombosis, which was absent when the mice were engrafted with PAD-4-deficient Jak2^V617F^ bone marrow. The Jak2^V617F^ mice treated with DNase 1 or the JAK inhibitor Ruxolitinib had a reduced thrombus size when subjected to the IVC stenosis model for DVT. Taken together, these findings implicated a mechanistic role of NETs in cancer-associated ischemic thrombosis.

### 3.3. NETosis and Age in the Context of COVID-19

NETosis is by far the major underlying pathway observed in COVID-19-associated thrombosis [46,85,86,87,88,89]. Age may exert a profound influence on the intricate dynamics of the immune response during COVID-19 [90,91]. Understanding this relationship is vital because it offers valuable insights into why older individuals often experience more severe forms of COVID-19. As individuals age, their immune systems undergo several changes, including alterations in neutrophil function and a predisposition to chronic inflammation, creating an environment that may prime the immune response for heightened NETosis [92]. These age-related changes can result in a more robust NETosis response when confronted with viral infections like SARS-CoV-2. Consequently, the amplification of NETosis in older individuals can contribute to increased inflammation, tissue damage, and a more severe clinical course of COVID-19 [93]. We also reported the presence of NETs in the coronary clots removed from patients with COVID-19 [94]. Others have made similar observations for COVID-19 [95]. This interplay between age and NETosis in COVID-19 is an evolving area of research with significant implications for understanding disease pathogenesis and developing targeted therapeutic strategies.

## 4. NETosis in Non-ischemic Conditions

The role of NETs has also been implicated in several non-ischemic conditions that may involve vascular components. For example, emerging evidence suggests that NETosis may play a role in neuroinflammation and the progression of neurodegenerative diseases. The release of NET components in the brain microenvironment may exacerbate neuronal damage [96,97]. NETosis has also been implicated in the pathogenesis of chronic obstructive pulmonary disease (COPD), which is more prevalent in older individuals. Neutrophil infiltration and excessive NET formation in the lungs can contribute to chronic inflammation and tissue damage in the respiratory system [98]. Rheumatoid arthritis (RA) is an autoimmune disease that often manifests in older adults. NETosis is known to play a role in RA pathology by promoting autoantibody production and joint inflammation [99,100]. Neutrophils release NETs containing citrullinated antigens, which can trigger an autoimmune response [101,102]. NETosis has been associated with kidney inflammation and fibrosis, both of which are common features of chronic kidney disease (CKD). The chronic, low-grade inflammation seen in CKD may be exacerbated by NETs released in older individuals with kidney disease [103,104]. NETosis has been linked to bone resorption, a process central to the development of osteoporosis and a condition that becomes more prevalent with age. Neutrophil-mediated inflammation and NET formation can contribute to bone loss [105]. 

Overall, NETosis plays a pivotal role not only in fostering a prothrombotic environment but also in various non-ischemic vascular conditions. Therefore, it is imperative to elucidate the upstream pathways leading to NETosis to pave way for the development of novel pharmaceutical agents for age-associated ischemic and non-ischemic vascular disorders. 

## 5. NLRP3 Inflammasome: A Key Mediator of NETosis-Driven Thrombosis

In the previous sections, we described that the release of NETs plays a crucial role in age-associated ischemic disease conditions, and thus holds potential as a therapeutic target. The emerging line of evidence indicates the involvement of the NLRP3 inflammasome activation in NETosis, and thus may contribute towards the onset of thrombosis. Inflammasome is a multimeric protein complex that assembles and activates within the host, in response to specific pathogens, is recognized by pattern recognition receptors [106,107]. Their activation triggers the release of proinflammatory cytokines and, therefore, is considered as an important component of the innate immune system. A typical inflammasome consists of an adaptor protein (apoptosis-associated speck-like protein with a caspase recruitment domain (ASC)) and caspase-1 [108]. NLRP3 acts as the sensor where the carboxyl-terminal LRR domain is involved in stimuli recognition. Upon receiving an appropriate stimulus, different components start assembling and result in the zymogenic conversion of procaspase-1 to caspase-1, which in turn, activates the proinflammatory cytokines IL-1β and IL-18 [109,110,111]. The inflammasome-mediated activation of IL-1β and IL-18 leads to a quick highly inducible proinflammatory response, which is tightly regulated. Moreover, inflammasome activation can set off a provocative cell death pathway pyroptosis, which depends on the cleavage of gasdermin and inhibits the replication of intracellular microorganisms [112].

NLRP3 is the most extensively studied member of inflammasomes, which can be activated by TLR ligands such as the LPS and tumor necrosis factor α (TNF-α). The precise mechanisms leading to the activation of the NLRP3 inflammasome are still debatable, besides being activated in response to wide variety of stimuli [113]. In monocytes, and macrophages, NLRP3 inflammasome activation requires a pre-treatment, or priming, using microbial-associated PAMPs or cytokines [114]. However, the NLRP3 inflammasome activation in platelets does not require any pre-treatment as the required components are constitutively expressed [115]. The activation of the inflammasome is mediated by a nuclear factor kappa-light-chain-enhancer of an activated B cell (NF-κB) [116]. The NLRP3 inflammasome plays a significant role in mediating the host immune defense against several bacterial, fungal, and viral infections [117,118,119]. The uncontrolled activation of the NLRP3 inflammasome contributes towards the pathogenesis of several diseases, including autoimmune diseases, diabetes, gout, cryopyrin-associated periodic syndromes, and atherosclerosis [113,120].

**Figure 2:**
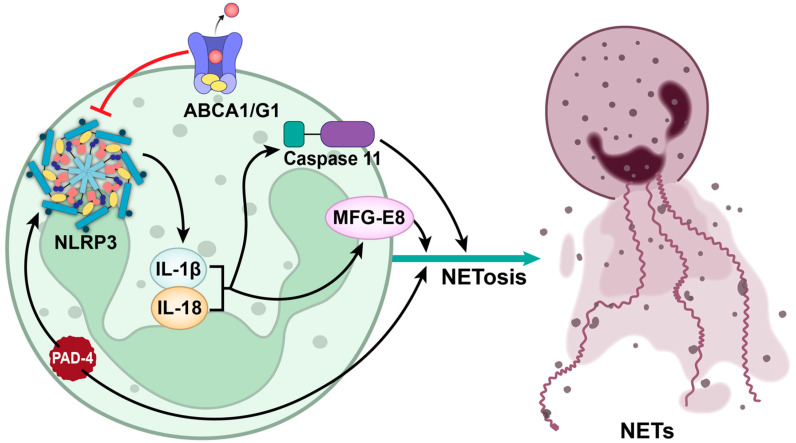
Interplay between NLRP3 inflammasome activation and NETosis in thrombotic disorders. This figure depicts the link between NLRP3 inflammasome activation and NETosis that may participate in ischemic thrombotic disorders. NLRP3 activation triggers NETosis, involving the release of prothrombotic NETs by the neutrophils. Notably, ABCA1/G1, caspase-11, IL-1β, IL-18, and MFG-E8 influence this interplay. ABCA1/G1 regulates NETosis through NLRP3 activation, while caspase-11 driven by IL-1β amplifies release of NETs. IL-18 can activate NETs and MFG-E8 contributes to IL-18-mediated NLRP3 activation. PAD-4 is essential for the release of NETs. However, PAD-4 also mediates the activation of the NLRP3 inflammasome, which in turn leads to the activation of proinflammatory cytokines, namely IL-18 and IL-1β. Together these cytokines promote the release of NETs via caspase-11 and MFG-8.

Kahlenberg et al. reported that NETs promote the activation of caspase-1 in macrophages, indicating an enhanced activity of NLRP3 [121]. Moreover, the same report suggested that IL-18, one of the byproducts of NLRP3 activation, exhibits the potential to activate NETs, and thus may suggest the presence of a bidirectional role of NLRP3 [121]. The results were further corroborated by Young et al., where they showed that IL-1β, another byproduct of NLRP3 activation, results in enhanced NETosis via caspase-11 activation [122]. Mechanistically, the IL-18-mediated activation of the NLRP3 inflammasome depends on the milk fat globule epidermal growth factor VIII (MFG-E8) [123]. In addition, the activation of NETosis via different stimuli may also require the presence of NLRP3 inflammasome activation (Figure 2). For example, Yalcinkaya et al. reported that the neutrophils specific deletion of the cholesterol transporters ATP-Binding Cassette A1 and G1 (ABCA1/G1) enhanced the extent of NETosis via the NLRP3 activation [124]. Furthermore, PAD-4, the enzyme necessary for the induction of NETosis, was also implicated in the NLRP3 inflammasome assembly [125]. Gasdermin D (GSDMD) is known to prevent an NET release via NLRP3 inflammasome inhibition [126]. A recent clinical study on COVID-19 reported that NLRP3 activation occurs much before NETs are released from the stimulated neutrophils, and hence validate their mechanistic involvement [127]. The genetic ablation of NLRP3 has been shown to result in a diminished NETs release [125]. Collectively these findings indicate that the NLRP3 inflammasome is a crucial upstream mediator for NET release. Hence, NLRP3 inhibition can alleviate the severity of pathological conditions, where the NET release contributes significantly.

Since NLRP3 activation plays an important role in the NET release, it is highly anticipated that their activation will contribute towards the onset of various thrombotic disorders. For example, antiphospholipid syndrome (APS) disorder, which is characterized by thromboembolic events and the presence of antiphospholipid antibodies [128,129], showed a dramatic increase in the NLRP3 activation in mononuclear cells isolated from patients with APS as well as from the murine model [130,131]. Similarly, rheumatic mitral stenosis (MS) patients with clinically relevant thrombosis exhibited higher levels of IL-1β when compared to the individuals who did not experienced thrombosis [132]. Notably, the expression was not restricted only to the immune cells. Murthy et al. reported the presence of NLRP3 in platelets and revealed their involvement in platelet activation and thrombosis, which was dependent on Bruton’s tyrosine kinase (BTK) [115]. Likewise, the pharmacological inhibition of NLRP3 or BTK using MCC950 or ibrutinib, respectively, can prevent sickle cell disease-associated platelet aggregation and activation [133]. However, these results were contradicted by another study, where it was reported that platelets did not express NLRP3 [134]. The latter publication claimed that the reason behind this discrepancy could be the difference in the methods used to determine the expression of NLRP3 [134]. While the former study evaluated the expression using immunofluorescence staining [115], the later used Western blotting [134]. Such discrepant results not merely raise methodological concerns but the need to develop robust technology that can be expanded for diagnostic purposes in patients.

IL-1β increases the susceptibility to arterial thrombosis by promoting the NETosis-dependent release of the tissue factor [135]. Interestingly, NLRP3 activation also contribute towards platelet activation and arterial thrombosis in vivo [115,136]. IL-β neutralization, using a specific monoclonal antibody canakinumabcan, can attenuate the severity of venous thrombosis in CD39-deficient mice [137]. The same group had previously shown that CD39 haploinsufficiency led to platelet hyperactivity and increased the risk for atherosclerosis [138,139,140]. Recently, Gupta et al. identified the NLRP3 inflammasome complex as a crucial determinant of hypoxia-mediated acute thrombotic events [141]. The NLRP3 inflammasome was recently also identified in subarachnoid hemorrhage (SAH)-dependent micro thrombosis [142]. These findings suggest an interplay of NLRP3 under various ischemic conditions, likely through NETs as the downstream mediator.

In a nutshell, NETosis plays an important role in the pathogenesis of thrombotic disorders through its upstream mediator, the NLRP3 inflammasome. Therefore, it is conceivable that the inhibition of NLRP3-mediated NETosis can pave the path for developing novel therapeutic approaches for several thrombotic disorders. 

### Inhibiting NLRP3: A Promising Therapeutic Approach for Thrombotic Disorders

Emerging evidence suggests that NLRP3 inhibition could be one of the anti-ischemic targets. NLRP3 inhibitors can be broadly categorized into synthetic and natural types. Natural compounds emerged as an alternative for developing therapeutics due to their enhanced bioavailability and minimal toxicity. Bromoxone is a potent marine natural compound that can inhibit the NLRP3 inflammasome activity with an IC_50_ value of 0.17 μM. Interestingly, the same compound can suppress the expression of NLRP3 via NFκB inhibition [143]. Artemisinin derived from the plant *Artemisia annua* exhibits a potent antimalarial activity [144], and can also attenuate the NLRP3 inflammasome assembly and subsequent activation [145]. Several other compounds, such as sulforaphane, a dietary isothiocyanate isolated from cruciferous vegetables such as broccoli; parthenopid, a plant-derived sesquiterpene lactone [146], and baicalin (BAI), a flavonoid compound isolated from *Scutellaria baicalensis* [147], may also be important NLRP3 inhibitors. However, these molecules have not been assessed for their NLRP3 inhibition-based protective role against thrombotic complications. 

Gegen Qinlian pills (GQPs) is a traditional Chinese medicine (TCM) that consists of four herbs, namely *Pueraria montana*, Scutellaria baicalensis, *Coptis chinensis*, and *Glycyrrhiza uralensis* [148]. Recently, Wei et al. reported that GQPs conferred protection against carrageenan-induced thrombosis in mice via NLRP3 inhibition [148]. Resveratrol, a polyphenol found in red wine, reduced the expression of NLRP3 and diminished the severity of venous thrombosis in a murine model [149]. Emerging clinical studies further strengthened the evidence supporting the protective impact of NLRP3 inhibition against thrombosis and cardiovascular diseases. The Canakinumab Anti-inflammatory Thrombosis Outcomes Study (CANTOS) demonstrated that targeting interleukin-1β (IL-1β) using canakinumabcan could significantly reduce the incidences of cardiovascular events [150,151,152]. A significant positive correlation was found between NLRP3 and troponin T in STEMI patients recruited under the ST-elevation myocardial infarction (TASTI) study [153].

Several synthetic compounds have been designed and developed that can inhibit the assembly and activation of the NLRP3 inflammasome. Refer to Table 1 for a detailed description of the compounds used to inhibit NLRP3. In 2001, a library of diarylsulfonylurea-containing compounds was screened for its ability to inhibit IL-1β processing [154]. Among them, MCC950 was identified as the most potent and selective inhibitor of the NLRP3 inflammasome with an IC_50_ value of 7.5 nM [155]. Harrison et al. developed novel ester-substitutes of MCC 950 and identified two compounds with a better inhibitory potential on IL-1β secretion in whole blood [156]. The inflammasome inhibitor MCC950 has been shown to reduce thrombosis in 4T1 tumor-bearing mice [83]. AMS-17, a novel sulfonylurea-derived compound, inhibited the NLRP3 inflammasome and prevented caspase-1 activation in the microglial cells upon stimulation with LPS [157]. JC124 is a benzenesulfonamide analogue that can inhibit NLRP3 with an IC50 value of 3.25 μM [158]. 

A series of α,β-unsaturated electrophilic warheads were developed by Cocco et al. Among them, compound 5 was found to be the most potent for inhibiting NLRP3 inflammasome-dependent pyroptosis [159]. Compound 5 was further modified using acrylamide functionality to develop 2-(2-chlorobenzyl)-*N*-(4-sulfamoylphenethyl) acrylamide (INF58), which inhibited the NLRP3 ATPase activity with an IC_50_ value of 74 μM [160]. It was observed that IFN58 interacted with Cys419 in the active site of NLRP3 to exhibit its inhibitory effect [160]. Using the structure-based pharmacophore modeling of triazolopyrimidinone, Harrison et al. (2022) developed NDT-30805, which inhibited the NLRP3 inflammasome with an IC_50_ value of 13 nM. Recently, a novel NLRP3 inhibitory compound 7 (NIC7) was designed by Haseeb et al. (2022) that inhibited the NLRP3 inflammasome with an IC_50_ of 9.0 μM [161]. Baldwin et al. used 2APB as a scaffold to develop NBC6 in order to reduce the non-specific effects on Ca^2+^ homeostasis. Moreover, in terms of potential, NBC6 performed better when compared to 2APB [162]. It was observed that the lipophilicity of the CCl3 group, not the substitutions on the aryl rings, played a key role in the enhanced activity of NBC6 [162]. The structure–activity relationship further suggested that it was key to the inhibitory activity of NBC6 and the substitutions on the aryl rings did not enhance the inhibitory potential [160]. These findings suggest the availability of several useful compounds for targeting NLRP3.

**Table 1:** Synthetic compounds inhibiting the NLRP3 inflammasome in various models.

Compound	Model	Results	IC_50_	Reference
Glyburide	Bone marrow-derived macrophages treated with LPS and ATP, and blood-derived monocytes treated with LPS and ATP	↓ secretion of mature IL-1β	12 μM (monocytes)	[154,163]
2-aminoethoxy diphenylborinate (2APB)	Peritoneal macrophages treated with LPS and ATP	↓ secretion of mature IL-1β	67 μM	[162]
EC144	Bone marrow-derived macrophages treated with nigericin and LPS	↓ formation of ASC specks	99 nM	[164]
MCC950	Bone marrow-derived macrophages stimulated with LPS and ATP	↓ secretion of mature IL-1β	7.5 nM	[165]
Compound 44 (ester-substitute of MCC 950)	Peripheral blood mononuclear cells stimulated with LPS and ATP	↓ secretion of mature IL-1β	36 nM	[156]
Compound 45 (ester-substitute of MCC 950)	Peripheral blood mononuclear cells stimulated with LPS and ATP	↓ secretion of mature IL-1β	30 nM	[156]
AMS-17	N9 microglial cells stimulated with LPS	↓ secretion of mature IL-1β	2.8 µM of AMS-17 reduced the IL-1β protein level by 30%	[157]
JC124	J774A.1 macrophage cells stimulated with LPS and ATP	↓ secretion of mature IL-1β	3.25 µM	[158]
Compound 5	THP-1 macrophage cells stimulated with LPS and ATP	↓ pyroptosis	10 µM of compound reduced pyroptosis by 100%	[159]
INF58	THP-1 macrophage cells stimulated with LPS and ATP	NLRP3 ATPase activity	74 μM	[160]
NDT-30805g	Peripheral blood mononuclear cells stimulated with LPS and ATP	↓ secretion of mature IL-1β	13 nM	[156]
NIC7w	THP-1 macrophage cells stimulated with LPS and nigericin	↓ secretion of mature IL-1β	9 µM	[161]
NBC6	Mouse peritoneal macrophages stimulated with LPS and ATP	↓ secretion of mature IL-1β	574 nM	[162]

## 6. Summary

Thrombotic ischemic complications, such as MI, stroke, DVT, and PE, have emerged as significant causes of morbidity and mortality in the elderly. Understanding the role of age and age-associated factors in thrombosis and incorporating this knowledge into clinical practice is vital for improving health outcomes in the elderly, and thereby addressing the growing health challenges associated with aging populations. This comprehensive review delves into the intricate relationship between age or the age-associated risk factors, NETs, and thrombosis with a primary focus on ischemic thrombotic disorders. Moreover, it underscores the importance of exploring NET inhibition and NLRP3 inhibition as potential therapeutic strategies and calls for further research to advance our understanding of these mechanisms and develop targeted interventions for age-associated ischemic thrombotic diseases. 

## References

[1] Wilkerson W.R., Sane D.C. (2002). Aging and thrombosis. Semin. Thromb. Hemost..

[2] Nurmohamed M.T., Büller H.R., ten Cate J.W. (1994). Physiological changes due to age. Implications for the prevention and treatment of thrombosis in older patients. Drugs Aging.

[3] Engbers M.J., van Hylckama Vlieg A., Rosendaal F.R. (2010). Venous thrombosis in the elderly: Incidence, risk factors and risk groups. J. Thromb. Haemost..

[4] Brinkmann V., Reichard U., Goosmann C., Fauler B., Uhlemann Y., Weiss D.S., Weinrauch Y., Zychlinsky A. (2004). Neutrophil extracellular traps kill bacteria. Science.

[5] Scharrig E., Carestia A., Ferrer M.F., Cedola M., Pretre G., Drut R., Picardeau M., Schattner M., Gomez R.M. (2015). Neutrophil Extracellular Traps are Involved in the Innate Immune Response to Infection with Leptospira. PLoS Negl. Trop. Dis..

[6] Fuchs T.A., Brill A., Duerschmied D., Schatzberg D., Monestier M., Myers D.D., Wrobleski S.K., Wakefield T.W., Hartwig J.H., Wagner D.D. (2010). Extracellular DNA traps promote thrombosis. Proc. Natl. Acad. Sci. USA.

[7] Stein P.D., Hull R.D., Kayali F., Ghali W.A., Alshab A.K., Olson R.E. (2004). Venous thromboembolism according to age: The impact of an aging population. Arch. Intern. Med..

[8] Anderson F.A., Spencer F.A. (2003). Risk factors for venous thromboembolism. Circulation.

[9] Muñoz-Torrero J.F., Bounameaux H., Pedrajas J.M., Lorenzo A., Rubio S., Kearon C., Hernández L., Monreal M. (2011). Effects of age on the risk of dying from pulmonary embolism or bleeding during treatment of deep vein thrombosis. J. Vasc. Surg..

[10] Gregson J., Kaptoge S., Bolton T., Pennells L., Willeit P., Burgess S., Bell S., Sweeting M., Rimm E.B., Kabrhel C. (2019). Cardiovascular Risk Factors Associated With Venous Thromboembolism. JAMA Cardiol..

[11] Brill A., Fuchs T.A., Savchenko A.S., Thomas G.M., Martinod K., De Meyer S.F., Bhandari A.A., Wagner D.D. (2012). Neutrophil extracellular traps promote deep vein thrombosis in mice. J. Thromb. Haemost..

[12] von Kockritz-Blickwede M., Goldmann O., Thulin P., Heinemann K., Norrby-Teglund A., Rohde M., Medina E. (2008). Phagocytosis-independent antimicrobial activity of mast cells by means of extracellular trap formation. Blood.

[13] Yousefi S., Gold J.A., Andina N., Lee J.J., Kelly A.M., Kozlowski E., Schmid I., Straumann A., Reichenbach J., Gleich G.J. (2008). Catapult-like release of mitochondrial DNA by eosinophils contributes to antibacterial defense. Nat. Med..

[14] Kumar R., Sonkar V.K., Swamy J., Ahmed A., Sharathkumar A.A., Pierce G.L., Dayal S. (2022). DNase 1 Protects From Increased Thrombin Generation and Venous Thrombosis During Aging: Cross-Sectional Study in Mice and Humans. J. Am. Heart Assoc..

[15] Dayal S., Wilson K.M., Motto D.G., Miller F.J., Chauhan A.K., Lentz S.R. (2013). Hydrogen peroxide promotes aging-related platelet hyperactivation and thrombosis. Circulation.

[16] Sonkar V.K., Eustes A.S., Ahmed A., Jensen M., Solanki M.V., Swamy J., Kumar R., Fidler T.P., Houtman J.C.D., Allen B.G. (2023). Endogenous SOD2 (Superoxide Dismutase) Regulates Platelet-Dependent Thrombin Generation and Thrombosis During Aging. Arter. Thromb. Vasc. Biol..

[17] Huang L., Li J., Jiang Y. (2016). Association between hypertension and deep vein thrombosis after orthopedic surgery: A meta-analysis. Eur. J. Med. Res..

[18] Kuller L.H., Velentgas P., Barzilay J., Beauchamp N.J., O’Leary D.H., Savage P.J. (2000). Diabetes mellitus: Subclinical cardiovascular disease and risk of incident cardiovascular disease and all-cause mortality. Arter. Thromb. Vasc. Biol..

[19] Wang J.C., Bennett M. (2012). Aging and atherosclerosis: Mechanisms, functional consequences, and potential therapeutics for cellular senescence. Circ. Res..

[20] Darvall K.A., Sam R.C., Silverman S.H., Bradbury A.W., Adam D.J. (2007). Obesity and thrombosis. Eur. J. Vasc. Endovasc. Surg..

[21] LaVasseur C., Neukam S., Kartika T., Samuelson Bannow B., Shatzel J., DeLoughery T.G. (2022). Hormonal therapies and venous thrombosis: Considerations for prevention and management. Res. Pr. Thromb. Haemost..

[22] Kunutsor S.K., Mäkikallio T.H., Seidu S., de Araújo C.G.S., Dey R.S., Blom A.W., Laukkanen J.A. (2020). Physical activity and risk of venous thromboembolism: Systematic review and meta-analysis of prospective cohort studies. Eur. J. Epidemiol..

[23] North B.J., Sinclair D.A. (2012). The intersection between aging and cardiovascular disease. Circ. Res..

[24] Njeim R., Azar W.S., Fares A.H., Azar S.T., Kfoury Kassouf H., Eid A.A. (2020). NETosis contributes to the pathogenesis of diabetes and its complications. J. Mol. Endocrinol..

[25] Wong S.L., Demers M., Martinod K., Gallant M., Wang Y., Goldfine A.B., Kahn C.R., Wagner D.D. (2015). Diabetes primes neutrophils to undergo NETosis, which impairs wound healing. Nat. Med..

[26] Takei H., Araki A., Watanabe H., Ichinose A., Sendo F. (1996). Rapid killing of human neutrophils by the potent activator phorbol 12-myristate 13-acetate (PMA) accompanied by changes different from typical apoptosis or necrosis. J. Leukoc. Biol..

[27] Wang Y., Li M., Stadler S., Correll S., Li P., Wang D., Hayama R., Leonelli L., Han H., Grigoryev S.A. (2009). Histone hypercitrullination mediates chromatin decondensation and neutrophil extracellular trap formation. J. Cell Biol..

[28] de Bont C.M., Boelens W.C., Pruijn G.J.M. (2019). NETosis, complement, and coagulation: A triangular relationship. Cell. Mol. Immunol..

[29] Martinod K., Demers M., Fuchs T.A., Wong S.L., Brill A., Gallant M., Hu J., Wang Y., Wagner D.D. (2013). Neutrophil histone modification by peptidylarginine deiminase 4 is critical for deep vein thrombosis in mice. Proc. Natl. Acad. Sci. USA.

[30] Martinod K., Witsch T., Farley K., Gallant M., Remold-O’Donnell E., Wagner D.D. (2016). Neutrophil elastase-deficient mice form neutrophil extracellular traps in an experimental model of deep vein thrombosis. J. Thromb. Haemost..

[31] Pertiwi K.R., de Boer O.J., Mackaaij C., Pabittei D.R., de Winter R.J., Li X., van der Wal A.C. (2019). Extracellular traps derived from macrophages, mast cells, eosinophils and neutrophils are generated in a time-dependent manner during atherothrombosis. J. Pathol..

[32] Schorn C., Janko C., Latzko M., Chaurio R., Schett G., Herrmann M. (2012). Monosodium urate crystals induce extracellular DNA traps in neutrophils, eosinophils, and basophils but not in mononuclear cells. Front. Immunol..

[33] Behzadifard M., Soleimani M. (2022). NETosis and SARS-COV-2 infection related thrombosis: A narrative review. Thromb. J..

[34] Ammollo C.T., Semeraro F., Xu J., Esmon N.L., Esmon C.T. (2011). Extracellular histones increase plasma thrombin generation by impairing thrombomodulin-dependent protein C activation. J. Thromb. Haemost..

[35] Urak K.T., Blanco G.N., Shubham S., Lin L.H., Dassie J.P., Thiel W.H., Chen Y., Sonkar V.K., Lei B., Murthy S. (2019). RNA inhibitors of nuclear proteins responsible for multiple organ dysfunction syndrome. Nat. Commun..

[36] Semeraro F., Ammollo C.T., Morrissey J.H., Dale G.L., Friese P., Esmon N.L., Esmon C.T. (2011). Extracellular histones promote thrombin generation through platelet-dependent mechanisms: Involvement of platelet TLR2 and TLR4. Blood.

[37] Vu T.T., Leslie B.A., Stafford A.R., Zhou J., Fredenburgh J.C., Weitz J.I. (2016). Histidine-rich glycoprotein binds DNA and RNA and attenuates their capacity to activate the intrinsic coagulation pathway. Thromb. Haemost..

[38] Bhagirath V.C., Dwivedi D.J., Liaw P.C. (2015). Comparison of the Proinflammatory and Procoagulant Properties of Nuclear, Mitochondrial, and Bacterial DNA. Shock.

[39] Gould T.J., Vu T.T., Swystun L.L., Dwivedi D.J., Mai S.H., Weitz J.I., Liaw P.C. (2014). Neutrophil extracellular traps promote thrombin generation through platelet-dependent and platelet-independent mechanisms. Arter. Thromb. Vasc. Biol..

[40] Longstaff C., Varju I., Sotonyi P., Szabo L., Krumrey M., Hoell A., Bota A., Varga Z., Komorowicz E., Kolev K. (2013). Mechanical stability and fibrinolytic resistance of clots containing fibrin, DNA, and histones. J. Biol. Chem..

[41] Varjú I., Longstaff C., Szabó L., Farkas Á.Z., Varga-Szabó V.J., Tanka-Salamon A., Machovich R., Kolev K. (2015). DNA, histones and neutrophil extracellular traps exert anti-fibrinolytic effects in a plasma environment. Thromb. Haemost..

[42] Ducroux C., Di Meglio L., Loyau S., Delbosc S., Boisseau W., Deschildre C., Ben Maacha M., Blanc R., Redjem H., Ciccio G. (2018). Thrombus Neutrophil Extracellular Traps Content Impair tPA-Induced Thrombolysis in Acute Ischemic Stroke. Stroke.

[43] Komissarov A.A., Florova G., Idell S. (2011). Effects of extracellular DNA on plasminogen activation and fibrinolysis. J. Biol. Chem..

[44] Noubouossie D.F., Whelihan M.F., Yu Y.B., Sparkenbaugh E., Pawlinski R., Monroe D.M., Key N.S. (2017). In vitro activation of coagulation by human neutrophil DNA and histone proteins but not neutrophil extracellular traps. Blood.

[45] Stakos D.A., Kambas K., Konstantinidis T., Mitroulis I., Apostolidou E., Arelaki S., Tsironidou V., Giatromanolaki A., Skendros P., Konstantinides S. (2015). Expression of functional tissue factor by neutrophil extracellular traps in culprit artery of acute myocardial infarction. Eur. Heart J..

[46] Skendros P., Mitsios A., Chrysanthopoulou A., Mastellos D.C., Metallidis S., Rafailidis P., Ntinopoulou M., Sertaridou E., Tsironidou V., Tsigalou C. (2020). Complement and tissue factor-enriched neutrophil extracellular traps are key drivers in COVID-19 immunothrombosis. J. Clin. Invest..

[47] Etulain J., Martinod K., Wong S.L., Cifuni S.M., Schattner M., Wagner D.D. (2015). P-selectin promotes neutrophil extracellular trap formation in mice. Blood.

[48] Constantinescu-Bercu A., Grassi L., Frontini M., Salles C., Woollard K., Crawley J.T. (2020). Activated alpha(IIb)beta(3) on platelets mediates flow-dependent NETosis via SLC44A2. Elife.

[49] Apipongrat D., Numbenjapon T., Prayoonwiwat W., Arnutti P., Nathalang O. (2019). Association between SLC44A2 rs2288904 polymorphism and risk of recurrent venous thromboembolism among Thai patients. Thromb. Res..

[50] Germain M., Chasman D.I., de Haan H., Tang W., Lindström S., Weng L.C., de Andrade M., de Visser M.C., Wiggins K.L., Suchon P. (2015). Meta-analysis of 65,734 individuals identifies TSPAN15 and SLC44A2 as two susceptibility loci for venous thromboembolism. Am. J. Hum. Genet..

[51] Hinds D.A., Buil A., Ziemek D., Martinez-Perez A., Malik R., Folkersen L., Germain M., Malarstig A., Brown A., Soria J.M. (2016). Genome-wide association analysis of self-reported events in 6135 individuals and 252 827 controls identifies 8 loci associated with thrombosis. Hum Mol Genet.

[52] Caudrillier A., Kessenbrock K., Gilliss B.M., Nguyen J.X., Marques M.B., Monestier M., Toy P., Werb Z., Looney M.R. (2012). Platelets induce neutrophil extracellular traps in transfusion-related acute lung injury. J. Clin. Invest..

[53] Clark S.R., Ma A.C., Tavener S.A., McDonald B., Goodarzi Z., Kelly M.M., Patel K.D., Chakrabarti S., McAvoy E., Sinclair G.D. (2007). Platelet TLR4 activates neutrophil extracellular traps to ensnare bacteria in septic blood. Nat. Med..

[54] Sabbatini M., Bona E., Novello G., Migliario M., Renò F. (2022). Aging hampers neutrophil extracellular traps (NETs) efficacy. Aging Clin. Exp. Res..

[55] Martinod K., Witsch T., Erpenbeck L., Savchenko A., Hayashi H., Cherpokova D., Gallant M., Mauler M., Cifuni S.M., Wagner D.D. (2017). Peptidylarginine deiminase 4 promotes age-related organ fibrosis. J. Exp. Med..

[56] Ortmann W., Kolaczkowska E. (2018). Age is the work of art? Impact of neutrophil and organism age on neutrophil extracellular trap formation. Cell Tissue Res..

[57] Matsuda Y., Itabashi M., Tachibana Y., Sugihara T., Sakashita Y., Matsubara T., Murayama S., Yumura W., Shimizu A., Takei T. (2019). Citrullinated histone H3 expression in anti-neutrophil cytoplasmic antibody-associated vasculitis in older Japanese autopsy patients. Geriatr. Gerontol. Int..

[58] Matsuda Y., Hamayasu H., Seki A., Nonaka K., Wang T., Matsumoto T., Hamano Y., Sumikura H., Kumasaka T., Murayama S. (2016). Presence of Citrullinated Histone H3-Positive Neutrophils in Microscopic Polyangiitis from the Early Phase: An Autopsy Proven Case. Pathol. Int..

[59] Shah M., He Z., Rauf A., Beikoghli Kalkhoran S., Heiestad C.M., Stenslokken K.O., Parish C.R., Soehnlein O., Arjun S., Davidson S.M. (2022). Extracellular histones are a target in myocardial ischaemia-reperfusion injury. Cardiovasc. Res..

[60] De Meyer S.F., Suidan G.L., Fuchs T.A., Monestier M., Wagner D.D. (2012). Extracellular chromatin is an important mediator of ischemic stroke in mice. Arter. Thromb. Vasc. Biol..

[61] Valles J., Lago A., Santos M.T., Latorre A.M., Tembl J.I., Salom J.B., Nieves C., Moscardo A. (2017). Neutrophil extracellular traps are increased in patients with acute ischemic stroke: Prognostic significance. Thromb. Haemost..

[62] Demyanets S., Stojkovic S., Mauracher L.M., Kopp C.W., Wojta J., Thaler J., Panzer S., Gremmel T. (2020). Surrogate Markers of Neutrophil Extracellular Trap Formation are Associated with Ischemic Outcomes and Platelet Activation after Peripheral Angioplasty and Stenting. J. Clin. Med..

[63] Fuchs T.A., Brill A., Wagner D.D. (2012). Neutrophil extracellular trap (NET) impact on deep vein thrombosis. Arter. Thromb. Vasc. Biol..

[64] Borissoff J.I., Joosen I.A., Versteylen M.O., Brill A., Fuchs T.A., Savchenko A.S., Gallant M., Martinod K., Ten Cate H., Hofstra L. (2013). Elevated levels of circulating DNA and chromatin are independently associated with severe coronary atherosclerosis and a prothrombotic state. Arter. Thromb. Vasc. Biol..

[65] Franck G., Mawson T.L., Folco E.J., Molinaro R., Ruvkun V., Engelbertsen D., Liu X., Tesmenitsky Y., Shvartz E., Sukhova G.K. (2018). Roles of PAD4 and NETosis in Experimental Atherosclerosis and Arterial Injury: Implications for Superficial Erosion. Circ. Res..

[66] Wan W., Liu H., Long Y., Wan W., Li Q., Zhu W., Wu Y. (2021). The association between circulating neutrophil extracellular trap related biomarkers and retinal vein occlusion incidence: A case-control pilot study. Exp. Eye Res..

[67] Fukui Y., Kawashima M., Kawaguchi K., Takeuchi M., Hirata M., Kataoka T.R., Sakurai T., Kataoka M., Kanao S., Nakamoto Y. (2018). Granulocyte-colony-stimulating factor-producing metaplastic carcinoma of the breast with significant elevation of serum interleukin-17 and vascular endothelial growth factor levels. Int. Cancer Conf. J..

[68] Yang X., Liu F., Xu Z., Chen C., Wu X., Li G., Li J. (2005). Expression of granulocyte colony stimulating factor receptor in human colorectal cancer. Postgrad. Med. J..

[69] Uematsu T., Tsuchie K., Ukai K., Kimoto E., Funakawa T., Mizuno R. (1996). Granulocyte-colony stimulating factor produced by pancreatic carcinoma. Int. J. Pancreatol..

[70] Kowanetz M., Wu X., Lee J., Tan M., Hagenbeek T., Qu X., Yu L., Ross J., Korsisaari N., Cao T. (2010). Granulocyte-colony stimulating factor promotes lung metastasis through mobilization of Ly6G+Ly6C+ granulocytes. Proc. Natl. Acad. Sci. USA.

[71] Jiang X., Lopez A., Holyoake T., Eaves A., Eaves C. (1999). Autocrine production and action of IL-3 and granulocyte colony-stimulating factor in chronic myeloid leukemia. Proc. Natl. Acad. Sci. USA.

[72] Demers M., Krause D.S., Schatzberg D., Martinod K., Voorhees J.R., Fuchs T.A., Scadden D.T., Wagner D.D. (2012). Cancers predispose neutrophils to release extracellular DNA traps that contribute to cancer-associated thrombosis. Proc. Natl. Acad. Sci. USA.

[73] Mauracher L.M., Posch F., Martinod K., Grilz E., Daullary T., Hell L., Brostjan C., Zielinski C., Ay C., Wagner D.D. (2018). Citrullinated histone H3, a biomarker of neutrophil extracellular trap formation, predicts the risk of venous thromboembolism in cancer patients. J. Thromb. Haemost..

[74] Guy A., Favre S., Labrouche-Colomer S., Deloison L., Gourdou-Latyszenok V., Renault M.A., Riviere E., James C. (2019). High circulating levels of MPO-DNA are associated with thrombosis in patients with MPN. Leukemia.

[75] Thalin C., Demers M., Blomgren B., Wong S.L., von Arbin M., von Heijne A., Laska A.C., Wallen H., Wagner D.D., Aspberg S. (2016). NETosis promotes cancer-associated arterial microthrombosis presenting as ischemic stroke with troponin elevation. Thromb. Res..

[76] Oklu R., Sheth R.A., Wong K.H.K., Jahromi A.H., Albadawi H. (2017). Neutrophil extracellular traps are increased in cancer patients but does not associate with venous thrombosis. Cardiovasc. Diagn. Ther..

[77] Kumar R., Katare P.B., Lentz S.R., Modi A.J., Sharathkumar A.A., Dayal S. (2021). Thrombotic potential during pediatric acute lymphoblastic leukemia induction: Role of cell-free DNA. Res. Pr. Thromb. Haemost..

[78] Hisada Y., Grover S.P., Maqsood A., Houston R., Ay C., Noubouossie D.F., Cooley B.C., Wallen H., Key N.S., Thalin C. (2019). Neutrophils and neutrophil extracellular traps enhance venous thrombosis in mice bearing human pancreatic tumors. Haematologica.

[79] Leal A.C., Mizurini D.M., Gomes T., Rochael N.C., Saraiva E.M., Dias M.S., Werneck C.C., Sielski M.S., Vicente C.P., Monteiro R.Q. (2017). Tumor-Derived Exosomes Induce the Formation of Neutrophil Extracellular Traps: Implications For The Establishment of Cancer-Associated Thrombosis. Sci. Rep..

[80] Varady C.B.S., Oliveira A.C., Monteiro R.Q., Gomes T. (2021). Recombinant human DNase I for the treatment of cancer-associated thrombosis: A pre-clinical study. Thromb. Res..

[81] Gomes T., Varady C.B.S., Lourenco A.L., Mizurini D.M., Rondon A.M.R., Leal A.C., Goncalves B.S., Bou-Habib D.C., Medei E., Monteiro R.Q. (2019). IL-1beta Blockade Attenuates Thrombosis in a Neutrophil Extracellular Trap-Dependent Breast Cancer Model. Front. Immunol..

[82] Wolach O., Sellar R.S., Martinod K., Cherpokova D., McConkey M., Chappell R.J., Silver A.J., Adams D., Castellano C.A., Schneider R.K. (2018). Increased neutrophil extracellular trap formation promotes thrombosis in myeloproliferative neoplasms. Sci. Transl. Med..

[83] Zuo Y., Estes S.K., Ali R.A., Gandhi A.A., Yalavarthi S., Shi H., Sule G., Gockman K., Madison J.A., Zuo M. (2020). Prothrombotic autoantibodies in serum from patients hospitalized with COVID-19. Sci. Transl. Med..

[84] Zuo Y., Yalavarthi S., Shi H., Gockman K., Zuo M., Madison J.A., Blair C., Weber A., Barnes B.J., Egeblad M. (2020). Neutrophil extracellular traps in COVID-19. JCI Insight.

[85] Zuo Y., Zuo M., Yalavarthi S., Gockman K., Madison J.A., Shi H., Woodard W., Lezak S.P., Lugogo N.L., Knight J.S. (2021). Neutrophil extracellular traps and thrombosis in COVID-19. J. Thromb. Thrombolysis.

[86] Middleton E.A., He X.Y., Denorme F., Campbell R.A., Ng D., Salvatore S.P., Mostyka M., Baxter-Stoltzfus A., Borczuk A.C., Loda M. (2020). Neutrophil extracellular traps contribute to immunothrombosis in COVID-19 acute respiratory distress syndrome. Blood.

[87] Ng H., Havervall S., Rosell A., Aguilera K., Parv K., von Meijenfeldt F.A., Lisman T., Mackman N., Thalin C., Phillipson M. (2021). Circulating Markers of Neutrophil Extracellular Traps Are of Prognostic Value in Patients With COVID-19. Arter. Thromb. Vasc. Biol..

[88] Janiuk K., Jabłońska E., Garley M. (2021). Significance of NETs Formation in COVID-19. Cells.

[89] Yaqinuddin A., Kvietys P., Kashir J. (2020). COVID-19: Role of neutrophil extracellular traps in acute lung injury. Respir. Investig..

[90] Li X., Li C., Zhang W., Wang Y., Qian P., Huang H. (2023). Inflammation and aging: Signaling pathways and intervention therapies. Signal Transduct. Target. Ther..

[91] Zhu Y., Chen X., Liu X. (2022). NETosis and Neutrophil Extracellular Traps in COVID-19: Immunothrombosis and Beyond. Front. Immunol..

[92] Hanna E.B., Rossen J., Eustes A.S., Dayal S. (2022). Heavy lone coronary artery thrombosis treated by stent retriever, in the setting of COVID-19 infection. Catheter. Cardiovasc. Interv..

[93] Schurink B., Roos E., Radonic T., Barbe E., Bouman C.S.C., de Boer H.H., de Bree G.J., Bulle E.B., Aronica E.M., Florquin S. (2020). Viral presence and immunopathology in patients with lethal COVID-19: A prospective autopsy cohort study. Lancet Microbe.

[94] Morgan W.H., Hazelton M.L., Yu D.Y. (2016). Retinal venous pulsation: Expanding our understanding and use of this enigmatic phenomenon. Prog. Retin. Eye Res..

[95] Freund K.B., Sarraf D., Leong B.C.S., Garrity S.T., Vupparaboina K.K., Dansingani K.K. (2018). Association of Optical Coherence Tomography Angiography of Collaterals in Retinal Vein Occlusion With Major Venous Outflow Through the Deep Vascular Complex. JAMA Ophthalmol..

[96] Pietronigro E.C., Della Bianca V., Zenaro E., Constantin G. (2017). NETosis in Alzheimer’s Disease. Front. Immunol..

[97] Byun D.J., Lee J., Yu J.W., Hyun Y.M. (2023). NLRP3 Exacerbate NETosis-Associated Neuroinflammation in an LPS-Induced Inflamed Brain. Immune Netw..

[98] Grabcanovic-Musija F., Obermayer A., Stoiber W., Krautgartner W.D., Steinbacher P., Winterberg N., Bathke A.C., Klappacher M., Studnicka M. (2015). Neutrophil extracellular trap (NET) formation characterises stable and exacerbated COPD and correlates with airflow limitation. Respir. Res..

[99] Lee K.H., Kronbichler A., Park D.D., Park Y., Moon H., Kim H., Choi J.H., Choi Y., Shim S., Lyu I.S. (2017). Neutrophil extracellular traps (NETs) in autoimmune diseases: A comprehensive review. Autoimmun. Rev..

[100] Khandpur R., Carmona-Rivera C., Vivekanandan-Giri A., Gizinski A., Yalavarthi S., Knight J.S., Friday S., Li S., Patel R.M., Subramanian V. (2013). NETs are a source of citrullinated autoantigens and stimulate inflammatory responses in rheumatoid arthritis. Sci. Transl. Med..

[101] Song W., Ye J., Pan N., Tan C., Herrmann M. (2020). Neutrophil Extracellular Traps Tied to Rheumatoid Arthritis: Points to Ponder. Front. Immunol..

[102] Corsiero E., Pratesi F., Prediletto E., Bombardieri M., Migliorini P. (2016). NETosis as Source of Autoantigens in Rheumatoid Arthritis. Front. Immunol..

[103] Salazar-Gonzalez H., Zepeda-Hernandez A., Melo Z., Saavedra-Mayorga D.E., Echavarria R. (2019). Neutrophil Extracellular Traps in the Establishment and Progression of Renal Diseases. Medicina.

[104] Tong S., Yang S., Li T., Gao R., Hu J., Luo T., Qing H., Zhen Q., Hu R., Li X. (2019). Role of neutrophil extracellular traps in chronic kidney injury induced by bisphenol-A. J. Endocrinol..

[105] Hajishengallis G., Moutsopoulos N.M., Hajishengallis E., Chavakis T. (2016). Immune and regulatory functions of neutrophils in inflammatory bone loss. Semin. Immunol..

[106] Broz P., Dixit V.M. (2016). Inflammasomes: Mechanism of assembly, regulation and signalling. Nat. Rev. Immunol..

[107] Takeuchi O., Akira S. (2010). Pattern recognition receptors and inflammation. Cell.

[108] Schroder K., Tschopp J. (2010). The inflammasomes. Cell.

[109] Latz E., Xiao T.S., Stutz A. (2013). Activation and regulation of the inflammasomes. Nat. Rev. Immunol..

[110] de Zoete M.R., Flavell R.A. (2013). Interactions between Nod-Like Receptors and Intestinal Bacteria. Front. Immunol..

[111] Strowig T., Henao-Mejia J., Elinav E., Flavell R. (2012). Inflammasomes in health and disease. Nature.

[112] Shi J., Zhao Y., Wang K., Shi X., Wang Y., Huang H., Zhuang Y., Cai T., Wang F., Shao F. (2015). Cleavage of GSDMD by inflammatory caspases determines pyroptotic cell death. Nature.

[113] Guo H., Callaway J.B., Ting J.P. (2015). Inflammasomes: Mechanism of action, role in disease, and therapeutics. Nat. Med..

[114] He Y., Hara H., Núñez G. (2016). Mechanism and Regulation of NLRP3 Inflammasome Activation. Trends Biochem. Sci..

[115] Murthy P., Durco F., Miller-Ocuin J.L., Takedai T., Shankar S., Liang X., Liu X., Cui X., Sachdev U., Rath D. (2017). The NLRP3 inflammasome and bruton’s tyrosine kinase in platelets co-regulate platelet activation, aggregation, and in vitro thrombus formation. Biochem. Biophys. Res. Commun..

[116] Bauernfeind F.G., Horvath G., Stutz A., Alnemri E.S., MacDonald K., Speert D., Fernandes-Alnemri T., Wu J., Monks B.G., Fitzgerald K.A. (2009). Cutting edge: NF-kappaB activating pattern recognition and cytokine receptors license NLRP3 inflammasome activation by regulating NLRP3 expression. J. Immunol..

[117] Allen I.C., Scull M.A., Moore C.B., Holl E.K., McElvania-TeKippe E., Taxman D.J., Guthrie E.H., Pickles R.J., Ting J.P. (2009). The NLRP3 inflammasome mediates in vivo innate immunity to influenza A virus through recognition of viral RNA. Immunity.

[118] Gross O., Poeck H., Bscheider M., Dostert C., Hannesschläger N., Endres S., Hartmann G., Tardivel A., Schweighoffer E., Tybulewicz V. (2009). Syk kinase signalling couples to the Nlrp3 inflammasome for anti-fungal host defence. Nature.

[119] Kanneganti T.D., Body-Malapel M., Amer A., Park J.H., Whitfield J., Franchi L., Taraporewala Z.F., Miller D., Patton J.T., Inohara N. (2006). Critical role for Cryopyrin/Nalp3 in activation of caspase-1 in response to viral infection and double-stranded RNA. J. Biol. Chem..

[120] Menu P., Vince J.E. (2011). The NLRP3 inflammasome in health and disease: The good, the bad and the ugly. Clin. Exp. Immunol..

[121] Kahlenberg J.M., Carmona-Rivera C., Smith C.K., Kaplan M.J. (2013). Neutrophil extracellular trap-associated protein activation of the NLRP3 inflammasome is enhanced in lupus macrophages. J. Immunol..

[122] Caution K., Young N., Robledo-Avila F., Krause K., Abu Khweek A., Hamilton K., Badr A., Vaidya A., Daily K., Gosu H. (2019). Caspase-11 Mediates Neutrophil Chemotaxis and Extracellular Trap Formation During Acute Gouty Arthritis Through Alteration of Cofilin Phosphorylation. Front. Immunol..

[123] Huang W., Jiao J., Liu J., Huang M., Hu Y., Ran W., Yan L., Xiong Y., Li M., Quan Z. (2020). MFG-E8 accelerates wound healing in diabetes by regulating “NLRP3 inflammasome-neutrophil extracellular traps” axis. Cell Death Discov..

[124] Yalcinkaya M., Fotakis P., Liu W., Endo-Umeda K., Dou H., Abramowicz S., Xiao T., Libby P., Wang N., Tall A.R. (2023). Cholesterol accumulation in macrophages drives NETosis in atherosclerotic plaques via IL-1β secretion. Cardiovasc. Res..

[125] Münzer P., Negro R., Fukui S., di Meglio L., Aymonnier K., Chu L., Cherpokova D., Gutch S., Sorvillo N., Shi L. (2021). NLRP3 Inflammasome Assembly in Neutrophils Is Supported by PAD4 and Promotes NETosis Under Sterile Conditions. Front. Immunol..

[126] Yang S., Feng Y., Chen L., Wang Z., Chen J., Ni Q., Guo X., Zhang L., Xue G. (2023). Disulfiram accelerates diabetic foot ulcer healing by blocking NET formation via suppressing the NLRP3/Caspase-1/GSDMD pathway. Transl. Res..

[127] Aymonnier K., Ng J., Fredenburgh L.E., Zambrano-Vera K., Münzer P., Gutch S., Fukui S., Desjardins M., Subramaniam M., Baron R.M. (2022). Inflammasome activation in neutrophils of patients with severe COVID-19. Blood Adv..

[128] Harper B.E., Wills R., Pierangeli S.S. (2011). Pathophysiological mechanisms in antiphospholipid syndrome. Int. J. Clin. Rheumtol.

[129] Wilson W.A., Gharavi A.E., Koike T., Lockshin M.D., Branch D.W., Piette J.C., Brey R., Derksen R., Harris E.N., Hughes G.R. (1999). International consensus statement on preliminary classification criteria for definite antiphospholipid syndrome: Report of an international workshop. Arthritis Rheum..

[130] Martirosyan A., Petrek M., Navratilova Z., Blbulyan A., Boyajyan A., Manukyan G. (2015). Differential regulation of proinflammatory mediators following LPS- and ATP-induced activation of monocytes from patients with antiphospholipid syndrome. Biomed. Res. Int..

[131] Müller-Calleja N., Köhler A., Siebald B., Canisius A., Orning C., Radsak M., Stein P., Mönnikes R., Lackner K.J. (2015). Cofactor-independent antiphospholipid antibodies activate the NLRP3-inflammasome via endosomal NADPH-oxidase: Implications for the antiphospholipid syndrome. Thromb. Haemost..

[132] He G., Tan W., Wang B., Chen J., Li G., Zhu S., Xie J., Xu B. (2016). Increased M1 Macrophages Infiltration Is Associated with Thrombogenesis in Rheumatic Mitral Stenosis Patients with Atrial Fibrillation. PLoS ONE.

[133] Vogel S., Kamimura S., Arora T., Smith M.L., Almeida L.E.F., Combs C.A., Thein S.L., Quezado Z.M.N. (2021). NLRP3 inflammasome and bruton tyrosine kinase inhibition interferes with upregulated platelet aggregation and in vitro thrombus formation in sickle cell mice. Biochem. Biophys. Res. Commun..

[134] Watanabe S., Usui-Kawanishi F., Komada T., Karasawa T., Kamata R., Yamada N., Kimura H., Dezaki K., Ohmori T., Takahashi M. (2020). ASC regulates platelet activation and contributes to thrombus formation independent of NLRP3 inflammasome. Biochem. Biophys. Res. Commun..

[135] Liberale L., Holy E.W., Akhmedov A., Bonetti N.R., Nietlispach F., Matter C.M., Mach F., Montecucco F., Beer J.H., Paneni F. (2019). Interleukin-1β Mediates Arterial Thrombus Formation via NET-Associated Tissue Factor. J. Clin. Med..

[136] Qiao J., Wu X., Luo Q., Wei G., Xu M., Wu Y., Liu Y., Li X., Zi J., Ju W. (2018). NLRP3 regulates platelet integrin αIIbβ3 outside-in signaling, hemostasis and arterial thrombosis. Haematologica.

[137] Yadav V., Chi L., Zhao R., Tourdot B.E., Yalavarthi S., Jacobs B.N., Banka A., Liao H., Koonse S., Anyanwu A.C. (2019). Ectonucleotidase tri(di)phosphohydrolase-1 (ENTPD-1) disrupts inflammasome/interleukin 1β-driven venous thrombosis. J. Clin. Invest..

[138] Hyman M.C., Petrovic-Djergovic D., Visovatti S.H., Liao H., Yanamadala S., Bouïs D., Su E.J., Lawrence D.A., Broekman M.J., Marcus A.J. (2009). Self-regulation of inflammatory cell trafficking in mice by the leukocyte surface apyrase CD39. J. Clin. Invest..

[139] Kanthi Y., Hyman M.C., Liao H., Baek A.E., Visovatti S.H., Sutton N.R., Goonewardena S.N., Neral M.K., Jo H., Pinsky D.J. (2015). Flow-dependent expression of ectonucleotide tri(di)phosphohydrolase-1 and suppression of atherosclerosis. J. Clin. Invest..

[140] Pinsky D.J., Broekman M.J., Peschon J.J., Stocking K.L., Fujita T., Ramasamy R., Connolly E.S., Huang J., Kiss S., Zhang Y. (2002). Elucidation of the thromboregulatory role of CD39/ectoapyrase in the ischemic brain. J. Clin. Invest..

[141] Gupta N., Sahu A., Prabhakar A., Chatterjee T., Tyagi T., Kumari B., Khan N., Nair V., Bajaj N., Sharma M. (2017). Activation of NLRP3 inflammasome complex potentiates venous thrombosis in response to hypoxia. Proc. Natl. Acad. Sci. USA.

[142] Zuo Y., He T., Liao P., Zhuang K., Yan X., Liu F. (2020). 17-Allylamino-Demethoxygeldanamycin Ameliorate Microthrombosis Via HSP90/RIP3/NLRP3 Pathway After Subarachnoid Hemorrhage in Rats. Acta Neurochir. Suppl..

[143] Gong Y.N., Wang X., Wang J., Yang Z., Li S., Yang J., Liu L., Lei X., Shao F. (2010). Chemical probing reveals insights into the signaling mechanism of inflammasome activation. Cell Res..

[144] Crespo-Ortiz M.P., Wei M.Q. (2012). Antitumor activity of artemisinin and its derivatives: From a well-known antimalarial agent to a potential anticancer drug. J. Biomed. Biotechnol..

[145] Shi J.Q., Zhang C.C., Sun X.L., Cheng X.X., Wang J.B., Zhang Y.D., Xu J., Zou H.Q. (2013). Antimalarial drug artemisinin extenuates amyloidogenesis and neuroinflammation in APPswe/PS1dE9 transgenic mice via inhibition of nuclear factor-κB and NLRP3 inflammasome activation. CNS Neurosci. Ther..

[146] Juliana C., Fernandes-Alnemri T., Wu J., Datta P., Solorzano L., Yu J.W., Meng R., Quong A.A., Latz E., Scott C.P. (2010). Anti-inflammatory compounds parthenolide and Bay 11-7082 are direct inhibitors of the inflammasome. J. Biol. Chem..

[147] Jin X., Liu M.Y., Zhang D.F., Zhong X., Du K., Qian P., Yao W.F., Gao H., Wei M.J. (2019). Baicalin mitigates cognitive impairment and protects neurons from microglia-mediated neuroinflammation via suppressing NLRP3 inflammasomes and TLR4/NF-κB signaling pathway. CNS Neurosci. Ther..

[148] Wei X., Zhang B., Wei F., Ding M., Luo Z., Han X., Tan X. (2022). Gegen Qinlian pills alleviate carrageenan-induced thrombosis in mice model by regulating the HMGB1/NF-κB/NLRP3 signaling. Phytomedicine.

[149] Fei J., Qin X., Ma H., Zhang X., Wang H., Han J., Yu C., Jiang J. (2022). Resveratrol Ameliorates Deep Vein Thrombosis-Induced Inflammatory Response Through Inhibiting HIF-1α/NLRP3 Pathway. Inflammation.

[150] Everett B.M., MacFadyen J.G., Thuren T., Libby P., Glynn R.J., Ridker P.M. (2020). Inhibition of Interleukin-1β and Reduction in Atherothrombotic Cardiovascular Events in the CANTOS Trial. J. Am. Coll. Cardiol..

[151] Ridker P.M., Everett B.M., Thuren T., MacFadyen J.G., Chang W.H., Ballantyne C., Fonseca F., Nicolau J., Koenig W., Anker S.D. (2017). Antiinflammatory Therapy with Canakinumab for Atherosclerotic Disease. N. Engl. J. Med..

[152] Ridker P.M., MacFadyen J.G., Thuren T., Libby P. (2020). Residual inflammatory risk associated with interleukin-18 and interleukin-6 after successful interleukin-1β inhibition with canakinumab: Further rationale for the development of targeted anti-cytokine therapies for the treatment of atherothrombosis. Eur. Heart J..

[153] Nordeng J., Schandiz H., Solheim S., Åkra S., Hoffman P., Roald B., Bendz B., Arnesen H., Helseth R., Seljeflot I. (2021). The Inflammasome Signaling Pathway Is Actively Regulated and Related to Myocardial Damage in Coronary Thrombi from Patients with STEMI. Mediat. Inflamm..

[154] Perregaux D.G., McNiff P., Laliberte R., Hawryluk N., Peurano H., Stam E., Eggler J., Griffiths R., Dombroski M.A., Gabel C.A. (2001). Identification and characterization of a novel class of interleukin-1 post-translational processing inhibitors. J. Pharmacol. Exp. Ther..

[155] Coll R.C., Robertson A.A., Chae J.J., Higgins S.C., Muñoz-Planillo R., Inserra M.C., Vetter I., Dungan L.S., Monks B.G., Stutz A. (2015). A small-molecule inhibitor of the NLRP3 inflammasome for the treatment of inflammatory diseases. Nat. Med..

[156] Harrison D., Boutard N., Brzozka K., Bugaj M., Chmielewski S., Cierpich A., Doedens J.R., Fabritius C.R.Y., Gabel C.A., Galezowski M. (2020). Discovery of a series of ester-substituted NLRP3 inflammasome inhibitors. Bioorg Med. Chem. Lett..

[157] Zhang C., Sajith A.M., Xu X., Jiang J., Phillip Bowen J., Kulkarni A., Hao J. (2022). Targeting NLRP3 signaling by a novel-designed sulfonylurea compound for inhibition of microglial inflammation. Bioorg Med. Chem..

[158] Fulp J., He L., Toldo S., Jiang Y., Boice A., Guo C., Li X., Rolfe A., Sun D., Abbate A. (2018). Structural Insights of Benzenesulfonamide Analogues as NLRP3 Inflammasome Inhibitors: Design, Synthesis, and Biological Characterization. J. Med. Chem..

[159] Cocco M., Garella D., Di Stilo A., Borretto E., Stevanato L., Giorgis M., Marini E., Fantozzi R., Miglio G., Bertinaria M. (2014). Electrophilic warhead-based design of compounds preventing NLRP3 inflammasome-dependent pyroptosis. J. Med. Chem..

[160] Cocco M., Miglio G., Giorgis M., Garella D., Marini E., Costale A., Regazzoni L., Vistoli G., Orioli M., Massulaha-Ahmed R. (2016). Design, Synthesis, and Evaluation of Acrylamide Derivatives as Direct NLRP3 Inflammasome Inhibitors. ChemMedChem.

[161] Haseeb M., Javaid N., Yasmeen F., Jeong U., Han J.H., Yoon J., Seo J.Y., Heo J.K., Shin H.C., Kim M.S. (2022). Novel Small-Molecule Inhibitor of NLRP3 Inflammasome Reverses Cognitive Impairment in an Alzheimer’s Disease Model. ACS Chem. Neurosci..

[162] Baldwin A.G., Rivers-Auty J., Daniels M.J.D., White C.S., Schwalbe C.H., Schilling T., Hammadi H., Jaiyong P., Spencer N.G., England H. (2017). Boron-Based Inhibitors of the NLRP3 Inflammasome. Cell Chem. Biol..

[163] Lamkanfi M., Mueller J.L., Vitari A.C., Misaghi S., Fedorova A., Deshayes K., Lee W.P., Hoffman H.M., Dixit V.M. (2009). Glyburide inhibits the Cryopyrin/Nalp3 inflammasome. J. Cell Biol..

[164] Nizami S., Arunasalam K., Green J., Cook J., Lawrence C.B., Zarganes-Tzitzikas T., Davis J.B., Di Daniel E., Brough D. (2021). Inhibition of the NLRP3 inflammasome by HSP90 inhibitors. Immunology.

[165] Dempsey C., Rubio Araiz A., Bryson K.J., Finucane O., Larkin C., Mills E.L., Robertson A.A.B., Cooper M.A., O’Neill L.A.J., Lynch M.A. (2017). Inhibiting the NLRP3 inflammasome with MCC950 promotes non-phlogistic clearance of amyloid-β and cognitive function in APP/PS1 mice. Brain Behav. Immun..

